# *FcStuA* from *Fusarium culmorum* Controls Wheat Foot and Root Rot in a Toxin Dispensable Manner

**DOI:** 10.1371/journal.pone.0057429

**Published:** 2013-02-22

**Authors:** Matias Pasquali, Francesca Spanu, Barbara Scherm, Virgilio Balmas, Lucien Hoffmann, Kim E. Hammond-Kosack, Marco Beyer, Quirico Migheli

**Affiliations:** 1 Environment and Agro-biotechnologies Department - Centre de Recherche Public - Gabriel Lippmann, Belvaux, Luxembourg; 2 Dipartimento di Agraria - Sezione di Patologia vegetale ed entomologia and Unità di ricerca Istituto Nazionale di Biostrutture e Biosistemi, Università degli Studi di Sassari, Sassari, Italy; 3 Wheat Pathogenomics, Plant Biology and Crop Science Department, Rothamsted Research, Harpenden, Herts, United Kingdom; 4 Centro interdisciplinare per lo sviluppo della ricerca biotecnologica e per lo studio della biodiversità della Sardegna e dell'area mediterranea, Università degli Studi di Sassari, Sassari, Italy; Seoul National University, Republic of Korea

## Abstract

*Fusarium culmorum* is one of the most harmful pathogens of durum wheat and is the causal agent of foot and root rot (FRR) disease. *F. culmorum* produces the mycotoxin deoxynivalenol (DON) that is involved in the pathogenic process. The role of the gene *FcStuA*, a StuA ortholog protein with an APSES domain sharing 98.5% homology to the *FgStuA* protein (FGSG10129), was determined by functional characterisation of deletion mutants obtained from two *F. culmorum* wild-type strains, FcUk99 (a highly pathogenic DON producer) and Fc233B (unable to produce toxin and with a mild pathogenic behavior). The *ΔFcStuA* mutants originating from both strains showed common phenotypic characters including stunted vegetative growth, loss of hydrophobicity of the mycelium, altered pigmentation, decreased activity of polygalacturonic enzymes and catalases, altered and reduced conidiation, delayed conidial germination patterns and complete loss of pathogenicity towards wheat stem base/root tissue. Glycolytic process efficiency [measured as growth on glucose as sole carbon (C) source] was strongly impaired and growth was partially restored on glutamic acid. Growth on pectin-like sources ranked in between glucose and glutamic acid with the following order (the lowest to the highest growth): beechwood xylan, sugarbeet arabinan, polygalacturonic acid, citrus pectin, apple pectin, potato azogalactan. DON production in the mutants originating from FcUK99 strain was significantly decreased (−95%) *in vitro*. Moreover, both sets of mutants were unable to colonise non-cereal plant tissues, i.e. apple and tomato fruits and potato tubers. No differences between mutants, ectopic and wild-type strains were observed concerning the level of resistance towards four fungicides belonging to three classes, the demethylase inhibitors epoxiconazole and tebuconzole, the succinate dehydrogenase inhibitor isopyrazam and the cytochrome bc1 inhibitor trifloxystrobin. *StuA*, given its multiple functions in cell regulation and pathogenicity control, is proposed as a potential target for novel disease management strategies.

## Introduction

In ascomycetous fungi, APSES proteins are an important class of transcription factors involved in the control of the main developmental processes and in the regulation of the cell-cycle. All members that belong to the family of APSES proteins have a ∼100-residue sequence-specific basic helix-loop-helix DNA-binding domain [1], [2], [3], [4]. APSES proteins are similar to viral KilA-N-domains implying probably a viral origin due to an ancestral infection of a fungal cell [5]. *Asm-1* (*Neurospora crassa* [6]), *Phd1* and *Sok2* (*Saccharomyces cerevisiae* [7]), *Efg1* and *Efh1* (*Candida albicans* [3]), *StuA* (*Aspergillus nidulans* [8]) are the main identified genes belonging to the APSES protein group. In *S*. *cerevisiae* and in *C*. *albicans* Phd1p, Sok2p, Efg1p, and Efh1p regulate genetic physiological and biochemical mechanisms responsible for dimorphic transition [7], [9], [10]. Asm-1 controls sexual and asexual reproduction. In general, StuA homologues regulate sporulation mechanisms, cellular differentiation, morphogenetic processes, mycelial growth, and virulence, but their role changes according to the fungal species.

*F. culmorum* is an important pathogen on cereals, distributed worldwide and able to produce a range of mycotoxins that are harmful to human and animal health [11], [12]. The main mycotoxins produced belong to the type B trichothecenes. Chemically, the trichothecenes are sesquiterpenoid compounds able to inhibit protein synthesis in eukaryotic cells and to induce apoptosis [13]. Moreover, the production of some trichothecenes, for example deoxynivalenol (DON), plays an important role during *Fusarium graminearum* infection of some host plant species, notably wheat floral tissue [14], [15], [16]. An analysis conducted by confocal laser scanner microscopy showed the infection processes and mechanisms of penetration by *F. culmorum* in foot and root rot diseases [17]. Initially, the fungal hyphae follow an apoplastic intercellular pathway and then colonise the cortex via a symplastic intercellular pathway. Nevertheless, *F. culmorum* does not penetrate into the root stele. Instead, section analysis of the stem showed that *F. culmorum* penetrates through the stomata of the leaf sheaths that wrap around the stem base [18].

Despite the economic importance of *F. culmorum* incited disease, knowledge on pathogenicity factors in *F. culmorum* is limited. Therefore, as a starting point, a study of the effect of potential key regulators in the genome is warranted. It has been already shown that DON production may play a role in wheat foot and root rot severity [19], [20]. Here we identified and characterised the role of FcStuA in *F. culmorum* aiming at understanding its role in foot and root rot and head blight pathogenicity, in the biosynthesis of pectolytic enzymes such as polygalacturonase and in the metabolic or morphological processes.

## Materials and Methods

### Strains and culture conditions

Transformation experiments were done with two *F. culmorum* wild-type strains: FcUK99 (Rothamsted Research, UK), a DON-producer (NRRL54111) isolated from an infected wheat plant [21], and Fc233B (NRRL54905), also isolated from an infected wheat plant [22], previously characterised as being unable to produce toxin *in vitro* (Pasquali *et al*., unpublished). Toxin production of strain Fc233B was verified according to the analytical procedure described by [22] in media derived from [23], (sugar source sucrose) and from [24], following cultural conditions as described by [25].

Morphological characterisation of wild-type and mutant strains was carried out on different substrates. Potato dextrose agar (PDA; Sigma-Aldrich, St. Louis, MO), synthetic low-nutrient agar medium (SNA, [26]) in weight/volume consisting of: 0.1% KH_2_PO_4_, 0.1% KNO_3_, 0.05% MgSO_4_ 7H_2_O, 0.05% KCl, 0.02%, glucose, 0.02% sucrose, and 2% agar, carboxy-methyl cellulose liquid medium (CMC, [27]), Czapek-Dox broth (C1551; Sigma-Aldrich) were used. Lyophilised mycelium for DNA extraction was obtained by growing fungi in liquid complete medium (CM) [28]. For polygalacturonase (PG) activity induction, the Szécsi medium was used [29]: NH_4_H_2_PO_4_ 0.09% (w/v), (NH_4_) 2HPO_4_ 0.2%, MgSO_4_ 7H_2_O 0.01%, KCl 0.05%, pectin ICN 1%. The cup plate analysis was done to evaluate polygalacturonase expression in 100 mM acetate buffer pH 4.0, 0.5% polygalacturonic acid, 0.8% agarose [30]. To compare the abilities of carbon (C) source utilisation, minimal medium (pH 6) containing as a sole C source 1% w/v one of the following compounds: xylan from beechwood, red arabinogalactan from sugar beet, pectin from citrus peel, pectin from apple, azo-galactan from potato, polygalacturonic acid (all from Sigma-Aldrich) were used as described in [31]. To verify the impairment of the glycolytic process in the mutants 30 mM of glucose or of glutamic acid monosodic (all media were adjusted to pH 6.0) was also used in the comparative growth test at 25 C in the dark. Growth was measured after 5 days on biological triplicates.

### Split-marker recombination and collection of *FcStuA* mutants

Conserved regions of the *StuA* gene in the three *Fusarium* species so far sequenced (*F. graminearum* PH-1, *Fusarium oxysporum* 4287 and *Fusarium verticillioides* 7600, available at: www.broadinstitute.org/annotation/genome/fusarium_group/MultiHome.html) were identified by *Muscle* alignments search done using the CLC Main Workbench v 6.01 software (CLC bio Aarhus, Denmark) aligning both DNA sequences and protein-derived sequences. The primers were designed by the same software to obtain the corresponding *StuA* gene sequence in *F. culmorum*. The upstream gene region was obtained from the genome sequencing project of *F. culmorum* strain UK99 (Hammond-Kosack, Antoniw, Urban *et al.*, unpublished). Split-marker recombination [32] was used for *FcStuA* deletion in FcUK99 and in Fc233B (**Fig. S1**) using the primers listed in **Table 1**.

**Table 1:** Primer sequences used to obtain the transforming constructs and to identify mutants by PCR and Southern blotting.

Split-marker recombination	Primer sequence
HY1F	5′-GGCTTGGCTGGAGCTAGTGGAGGTCAA-3′
HY2R	5′-GCCGAACCCGCTCGTCTGGCTAAGA-3′
YG1F	5′-GATGTAGGAGGGCGTGGATATGTCCT-3′
YG2R	5′-GAACCCGCGGTCGGCATCTACTCTAT-3′
StuA 1F	5′-CCGTTCTTAAACTTTGAAGCTCTATT-3′
StuA 2R	5′-TTGACCTCCACTAGCTCCAGCCAAGCCGAAAGCAGTCGTGATAAATGAAGAT-3′
StuA 3F	5′-GAATAGAGTAGATGCCGACCGCGGGTTCTATGCTTGCGAAATTGTAGATCAT-3′
StuA 4R	5′-GTGAGTCGAGGGAGTTACTGATTT-3′
Identification of mutants by PCR	Primer sequence
StuA NF	5′-CTTGATAAACGGCAGTGGAGA-3′
StuA NR	5′-GAATCTGTTCCGAACTCATCATT-3′
StuA 1F	5′-CCGTTCTTAAACTTTGAAGCTCTATT-3′
StuA 4R	5′-GTGAGTCGAGGGAGTTACTGATTT-3′
ITS1	5′-TCCGTAGGTGAACCTGCGG-3′
ITS4	5′-TCCTCCGCTTATTGATATGC-3′
Probes	Primer sequence
StuA NF	5′-CTTGATAAACGGCAGTGGAGA-3′
StuA NR	5′-GAATCTGTTCCGAACTCATCATT-3′

Fungal protoplasts were obtained from macroconidia germinated on PDA. Typically, 12–14 plates of PDA medium were overlaid with a disc of sterile cellophane (model 583 Gel Dryer; Bio-Rad Hercules, CA, USA) and inoculated with 10^6^ conidia of *F. culmorum* FcUK99 or Fc233B and incubated at 25°C for about 16–18 hours. Then, the young mycelium was scraped from the cellophane surface using a sterile spatula and transferred into 2 Petri dishes (90 mm of diameter) each one containing 10 mL of lysis solution consisting of 10 mg/mL of lysing enzymes (L1492, Sigma-Aldrich, St. Louis, MO, USA) dissolved in 1.2 M MgSO_4_ (pH 5.8).

After 3–4 hours of incubation at room temperature and slow (50–60 rpm) agitation, the protoplasts were purified according to [33] and used directly in fungal transformation as described by [34].

### Identification of *FcStuA* deletion mutants

To verify whether in the 20 transformants obtained from *F. culmorum* strains FcUK99 and Fc233B, *FcStuA* was correctly deleted by the *hph* gene (which confers resistance to hygromycin B), a screening on selective medium (PDA amended with 200 µg/mL of hygromycin B) was performed. Monosporic transformants were further checked by direct specific PCR analysis [35] and only 9 transformants (S2-S9-S11-S12-S13-S16-S17-S21-S22) from FcUK99 and two (S19–S20) from Fc233B were selected for Southern blot analysis. Primer sequences used are listed in **Table 1**.

Genomic DNA was obtained by a standard extraction method [36] from mycelium grown in 50–70 mL of CM at 25°C with gentle shaking (100–120 rpm) for 5 days. Then, the mycelium was filtered with Miracloth membrane (475855 Calbiochem, Merck Darmstadt, Germany) and freeze-dried for 3 days. For Southern blot analysis, a total of 5 µg of each genomic DNA was digested at 37°C for 16–18 hours with 50 units of *Eco*RV (New England Biolabs) in a final volume of 100 µL. Subsequently, digested DNA was separated by electrophoresis on a 1.2% agarose gel and transferred to a nylon membrane (Hybond-N Amersham Biosciences, GE Healthcare, USA) using a vacuum blotter (Model 785, Bio-Rad) according to standard procedures [37]. The membrane was then hybridised and the specific probes were detected with Dig High Prime DNA labelling kit and Detection Starter II (Roche Applied Science, Basel, Switzerland) as described in the manufacturer's protocols. To identify *FcStuA* gene deletion, the membrane was labelled by a left flank probe (806 bp) and after stripping the membrane was labelled by a partial gene *StuA* probe (401 bp).

The left flank probe was amplified using the TopTaq™ DNA Polymerase kit (QiagenS.p.A., Milan, Italy) in 100 µL of total volume containing: 10 µL of TopTaq™ DNA Polymerase amplification buffer, 0.2 mM dNTPs, 0.5 µM of primer stuA 1F, 0.5 µM of primer stuA 2R and 2.5 U of TopTaq™ DNA Polymerase, 50 ng of FcUK99 DNA. The reaction was done according to the following protocol: 94°C for 3 min, then 35 cycles of 94°C for 20 sec, 53°C for 20 sec and 72°C for 1 min, followed by a final elongation step at 72°C for 5 min. The partial *StuA* gene probe was obtained from DNA of FcUK99 as template with primers stuA NF-stuA NR and Phusion^®^ High-Fidelity PCR Master Mix with HF Buffer (New England Biolabs) at the following conditions: 98°C for 2 min, then 33 cycles of 98°C for 20 sec, 60°C for 30 sec and 72°C for 50 sec, followed by a final elongation step at 72°C for 5 min.

### Pathogenicity tests

*FcStuA* deletion mutants confirmed by Southern blot analysis were tested on durum wheat seedlings to evaluate the role of *FcStuA* in the pathogenic process by the soil-borne fungus *F. culmorum*.

Mycelium plugs bearing one seed of durum wheat (*Triticum durum* cv. Claudio, kindly provided by Unità di Ricerca per la Valorizzazione Qualitativa dei Cereali, CRA-QCE, Rome, Italy) were placed into a plastic sowing pot and covered by sterile soil. Pathogenicity tests were conducted according to [38] in a greenhouse at 25° C and three weeks after inoculation the severity of disease was assessed using the McKinney index [39].

Head Blight symptoms were evaluated on *Triticum durum* cv Simeto plants (kindly provided by CRA-QCE, Rome, Italy) inoculated according to the procedure described in [40]. Plants were observed every 3 days and final evaluation was carried out at 14 days after inoculation. Score indices ranged from 0 to 10, which is equivalent to the number of spikelets infected above the inoculation point.

In addition, the ability of *ΔFcStuA* mutants to colonise other plant tissues was evaluated. This included the inoculation of apple fruit slices [41], tomato fruits [42] and potato tuber slices with the respective wild-type and ectopic transformants. Thus, apple (cv. Golden Delicious), potato (cv. Spunta olandese) and tomato (cv. Altavilla) were washed under tap water and then disinfected within a sterile hood with a 2% sodium hypochlorite solution for 5 minutes and rinsed in sterile water at least twice. Later, plant tissues (except tomato) were cut into slices about 1 cm thick. Slices were placed in sterile Petri dishes and 10 µL of a concentrated spore suspension containing 10^5^ conidia/mL were pipetted on the surface. Inoculated samples were incubated in a dark room at 25°C and monitored daily for fungal growth. Tomato fruit were inoculated by direct deposition of the spore suspension on the fruit after creating a superficial damage with a pipetting tip. They were incubated in sterile plastic containers at the same modalities as described above. These experiments were performed three times.

### Microscopic observations of durum wheat kernels

To analyse the development of fungal hyphae during the first steps of colonisation, pathogenicity test conditions were reproduced *in vitro*. Ten mycelium discs (from the same set of strains as indicated before) bearing one seed were placed into a Petri dish and incubated 3 days in the dark.

The observations were conducted by a scanning electron microscope ZEISS EVO LS with environmental control in low vacuum conditions (pressure 600 Pa, temperature 2°C, humidity 85%). To investigate whether the impaired seedling germination was caused by seed death or by an inhibition of seed germination induced by surrounding fungal hyphae, seeds (washed with 1% NaClO 3 times for 1 minute, then washed with sterile water) were incubated in a water agar plate for 3 days in the dark after being in contact for three days with the mycelium. Germination of the seeds was then verified. The experiment was done in triplicate.

### Morphological analysis

From each of the *F. culmorum* FcUK99 and Fc233B wild-type strains, respectively, single-spore *FcStuA* deletion mutants (S12–S13 and S19) and an ectopic transformant (S2 and S20) were chosen for morphological characterisation. To evaluate mycelium hydrophobicity, a droplet (20 µL) of sterile H_2_O was deposited on the surface of colonies grown on PDA for 5 days. The time (in seconds) elapsed to absorb the drop by *FcStuA* deletion mutants and by ectopic transformants was compared to the respective wild-type strain. Hyphal structures, sporodochial development, conidiogenesis, as well as the time and extent of spore germination were observed by a light microscope (Olympus BX41) after growth in CMC liquid medium or SNA solid substrate. These experiments were replicated three times.

Briefly, three mycelium plugs were inoculated into 50–70 mL of liquid CMC medium in the dark at room temperature and 120 rpm shaking for 5 days. The spore suspension was filtered with a Miracloth® membrane and then washed twice with sterile water. The filtrate was centrifuged to collect conidia and subsequently the concentration was estimated using a Bürker-Türk hemacytometer. The growth experiment was done on SNA at 25 °C with a photoperiod of 24 hours for 18 days [43] to check sporodochial production. To determine the timing of spore germination, 1 mL of spore suspension (10^6^ conidia/mL) was inoculated into Erlenmeyer flasks containing 30 mL of Czapek-Dox broth. The evaluation was made after 0, 2, 4,and 8 h of incubation at 25 °C in the dark with slow shaking (100 rpm).

### Oxidative stress effects

To evaluate the ability of *FcStuA* mutants to grow in oxidative stress conditions, strains were cultured for 5 days at room temperature on CM plates amended with 3 mM H_2_O_2_, or with 50 mM potassium persulphate or with 10 mM methyl viologen. In addition, vegetative growth and pigment production were evaluated on PDA. Ten µL of a spore suspension (10^6^ conidia/mL) were spotted in the middle of each plate. After 5 days of incubation at room temperature the radial development was measured.

The effect of oxidative stress on the mutants was measured by comparing the ratios of growth (colony diameter at 5 days) of each strain to the respective wild type strain. A series of hydrogen peroxide concentrations (3- 4- 5- 6- 7 mM) were used to determine if the mutant and wild type fungal colonies differed in their sensitivity to high oxidative stress.

To analyse catalase activity in macroconidia, spores (1 ×10^5^ mL^−1^) of FcUK99. Fc233B and Δ*FgStuA* S12 and S19 mutants were suspended in 3.5 mM H_2_O_2_. The absorbance at 240 nm was measured every minute for 80 minutes with a Lambda 35 spectrophotometer (Perkin Elmer). The linear part of the degradation curve was compared and the resulting rate of degradation calculated as described in [45].

### Toxin analysis

The mycelium production and toxin inducing conditions were realised as previously described [25]. The test was conducted on FcUK99 wild-type, the ectopic S2 and the deletion mutants S12, S13. DON measurement was done using the AgraQuant^®^ DON test kit (Romerlab, Tulln, Austria) on medium after filtering out mycelium according to manufacturer's procedures. The absorbance of each well was determined at 630 and 450 nm using a Genious reader (Tecan Group Ltd., Männedorf, Switzerland).

To fit the toxin range sensitivity (i.e. the standard curve) of the kit, the wild-type and ectopic growth media were diluted in water 20 times. Values were calculated per g of dried mycelium mass. Three biological replicates were examined.

### “Cup-plate” analysis

The diffusion assay on agarose gel - referred to as “cup-plate” - allows a visual estimate of polygalacturonase (PG) content produced by fungi [30]. For this analysis the following strains were chosen: FcUK99, S12, S13, S2 and Fc233B, S19, S20. Five mycelium plugs (5 mm of diameter) for each strain were inoculated in a 250 mL Erlenmeyer flask with 50 mL of Szécsi medium [28] at 25°C with gentle shaking (100–120 rpm) for 3 days to induce PG activity. Then, the cultures were filtered by two layers of filter paper and 100 µL of solution were inoculated into the wells of 0.8 mm of diameter which had previously been made on the surface of the agarose plate. The agarose gel Petri dishes were incubated at 30°C for 20 hours in the dark. After incubation with 6–10 mL of 6N HCl for 15 minutes the plates were washed with distilled water. The enzyme activity was measured according to the size of the inhibition halo formed around the holes as a result of polygalacturonic matrix degradation by polygalacturonase enzymes.

### Fungicide sensitivity assays

The sensitivity of the wild type strains FcUK99 and Fc233B, of the two ectopics S2 and S20, as well as of the *FcStuA* deletion mutants S9, S12, S13 and S19 towards the fungicides epoxiconazole, isopyrazam, tebuconazole and trifloxystrobin was evaluated *in vitro* using the method described in [44].

### Statistical analysis

The descriptive statistics and the one-way ANOVA, followed by multiple comparisons applying the *post hoc* Tukey test, were done using the statistical analysis software package SPSS 19.0. We evaluated the percentage of seedling emergence, the disease severity, fungicides dose response curves, the vegetative growth on CM amended with oxidative stress inducers, and the polygalacturonase production. Whenever fungicides were able to inhibit fungal growth by more than 50%, the concentration inhibiting fungal growth by 50% (EC_50_) was calculated by fitting a curve
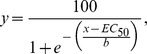



(where y  =  optical density of the fungal culture (% of fungicide free control), x = concentration of the fungicide (mM) and b  =  slope parameter).

The *ΔFcStuA* mutants and the ectopic co-transformants were compared with the respective wild-type strains as a control. In the case of toxin production, values were log transformed and significance was assayed using the Tukey test.

For carbon source effects, fungal colony diameter measurements after 5 days were subjected to a 2-factorial analysis of variance, where the different carbon sources in the medium was factor 1 and the fungal strain was factor 2. Subsequently, a multiple comparison procedure according to Tukey was applied.

The confidence level for all tests was set to 95%.

## Results

### Gene characteristics and mutant generation

The *FcStuA* gene sequence was obtained by amplification of FcUK99 DNA using conserved primers designed on the aligned *F. culmorum* genomic sequences available (**Fig. S2**). Manual inspection of the annotated *FcStuA* gene sequence revealed that the corresponding protein has a predicted 60.88 KDa weight with an isoelectric point of 8.71. It is composed of 553 amino acids, with 98.5% homology towards the FgStuA protein from *F. graminearum*.

By using the split marker PCR approach (**Fig. S1**), 20 hygromycin B-resistant transformants were obtained, 17 showing a *FcStuA* deletion and 3 being ectopic transformants. These PCR results were confirmed by Southern blot analysis of 11 randomly selected transformants. Overall, 16 *FcStuA* deletion mutants and 2 ectopic transformants were obtained from FcUK99, while one *FcStuA* deletion mutant and a single ectopic transformant were obtained from Fc233B.

### Deletion of *FcStuA* results in loss of pathogenicity in *Fusarium culmorum*

**Figure 1:**
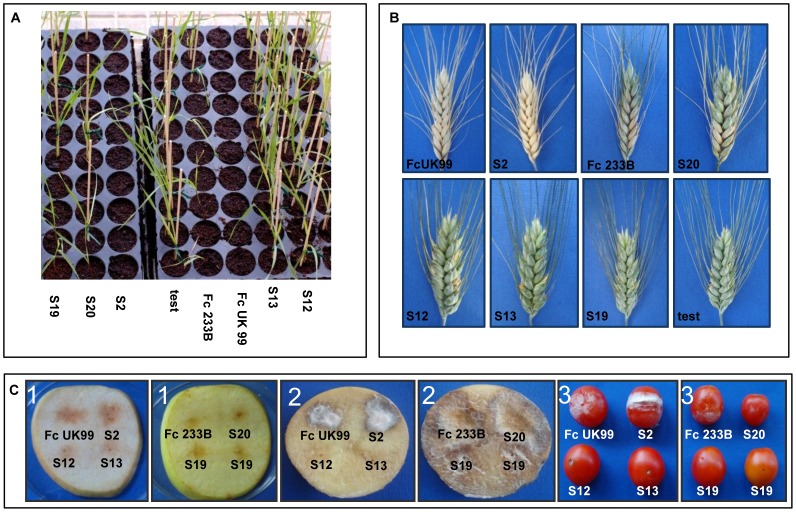
Pathogenicity of the *Fusarium culmorum* wild type and Δ*FcStuA* mutant strains and ectopic transformants on various plant species and tissue types. A: 21-d old durum wheat cv Claudio seedlings infected with Δ*FcStuA* mutants, wild types and ectopics and mock inoculated (test). No FRR symptoms were detected on the plants derived from the seeds inoculated with the mutants (S19, S20, S12 and S13). B: Spike of durum wheat cv Simeto infected with FcUK99, with an ectopic transformant or with the four independent Δ*FcStuA* mutants. No symptoms are visible on the mutant inoculated spikes. Photographs were taken 14 days post inoculation. C: Testing the ability for the same set of fungal strains to colonise three different plant tissues: apple (1), potato (2) and tomato (3). Cut or wounded surfaces were inoculated with *StuA* deleted mutants (S12, S13, S19), ectopic transformants (S2 and S20) or the wild-type strains (FcUK99 and Fc233B). Photographs were taken at either 4 days post inoculation (dpi) (apple and tomato) or 7 dpi (potato).

The pathogenicity of the wild-type strains FcUK99 and Fc233 B was confirmed to differ by 50% (**Table 2**, **Fig. 1**). The deletion of the *FcStuA* gene from both strains caused a complete impairment in pathogenicity. Statistical analysis indicated highly significant (*P*< 0.001) reduction of FRR symptoms on durum wheat seedlings for all 10 mutants tested, whilst the ectopic transformants did not differ significantly from the wild-type reference strains (**Table 2**; **Fig. 1A**). Also, in the floral infection tests, as previously reported in *F. graminearum* [45], the *FcStuA* deletion caused the complete absence of symptoms in the plant (**Fig. 1B**). Symptoms caused by the non-toxigenic strains Fc233B were very mild but significantly stronger than the respective mutant (**Fig. 1B**). In addition, the *FcStuA* deletion mutants showed a reduced ability to colonise fruit and tuber tissues of the non-cereal host plants, namely apple tomato and potato (**Fig. 1C**). Collectively, these results further strengthen the hypothesis that the disruption of the *FcStuA* gene has a major impact on fungal virulence in *F. culmorum*.

**Table 2:** Phenotypic measures of the strains used in this study.

	FRR severity^a^	FRR emergence^b^	FHB pathogenicity^c^	Hydrophobicity of the mycelium^d^	Conidia production at 8 days^e^	Conidia germination at 4 hrs^f^	Conidia germination at 8 hrs^g^	DON production in (µg per mg of dry weight)^h^	Polygalacturonic enzyme production^i^
Strain									
FcUK99 (wild type)	100.00±0.00	3.3±5.8	9.58 ±0.72	>60	10.02±2.39	73±6	96.33±2.05	6.49±3.33	20.59±1.27
S2 (ectopic)	100.00±0.00	0.0	8.66±1.21	>60	9.06±3.34	81±2.6	96.00±2.94	9.07±4.43	21.44±0.49
S12 (mutant)	10.00±10.00*	90.0±10.0*	0±0*	9	0.94±0.74*	41±5.7*	94.00±2.94	0.18±0.05*	17.31±0.98*
S13 (mutant)	10.00±10.00*	90.0±10.0*	0±0*	9	0.76±0.18*	55±2*	91.67±2.36	0.26±0.03*	17.13±1.32*
Fc233B (wild type)	70.83±15.07	43.3±15.3	3.14±0.75	5	5.47±1.02	93,3±3	93.00±2.83	Not measurable	28.85±2.62
S20 (ectopic)	69.2±19.4	53.3±15.3	2.58±0.22	13	7.86±1.82	95±2	88.67±6.34	Not measurable	28.46±2.07
S19(mutant)	6.67±5.77*	93.3±5.8*	0±0*	14	1.41±0.16*	49.7±2.9*	88.67±5.73	Not measurable	21.53±1.09*

Foot and root rot severity measured with McKinney index (0–100) ^a^ and emergence ^b^ tested on durum wheat cv Claudio; number of spikes infected at 14 days above the point of inoculation [38] ^c^ ; hydrophobicity of the mycelium measured in seconds needed before absorption of a 20 µ l water drop^d^; millions of conidia produced in CMC medium inoculated with 10^3^ conidia after 5 days growth^e^; percentage of germination of conidia after 4 hours on CM^f^; percentage of germinating conidia at 8 hrs (all mutants showed a delayed germination but no significant difference in the number of germinating conidia was observed)^g^; DON production in toxin inducing liquid medium corrected for mg of dry weight after 8 days culturing^h^; polygalacturonase enzyme activity measured with a diffusion assay on agarose gel (“cup plate”); measurements of pectolytic enzyme activity in diameter (mm) corrected by dry fungal biomass (mg)^i^. * P<0.01 by Tukey's post-hoc test.

All values are followed by SD. Significant differences compared to respective wild type are marked with an asterisk.

**Figure 2:**
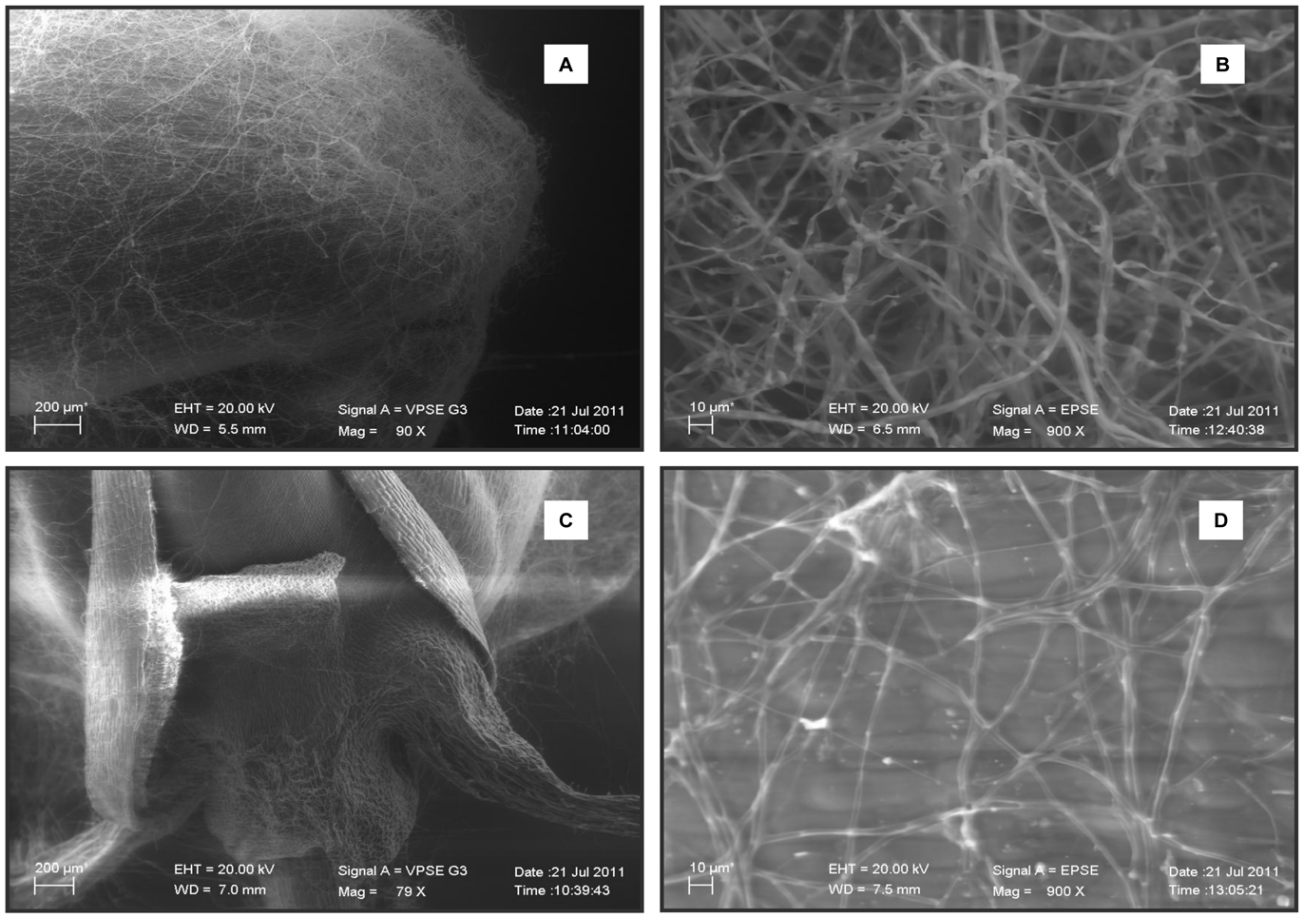
Scanning electron microscope analysis of the infection of durum wheat seeds by *Fusarium culmorum* wild-type and Δ*FcStuA* mutant strains. These ZEISS EVO LS images reveal the appearance of the surface of a seed of durum wheat placed over a mycelium disk of either wild-type *Fusarium culmorum* FcUK99 (A and B) or its ΔFc*StuA* mutant (C and D), 3 days after incubation at 25°C. During observation the samples were subjected to the following conditions: temperature 2°C, humidity 85% and pressure 600 Pa. A and C panel show an apical seed view. B and D show a detail of the mycelium surrounding the seed.

**Figure 3:**
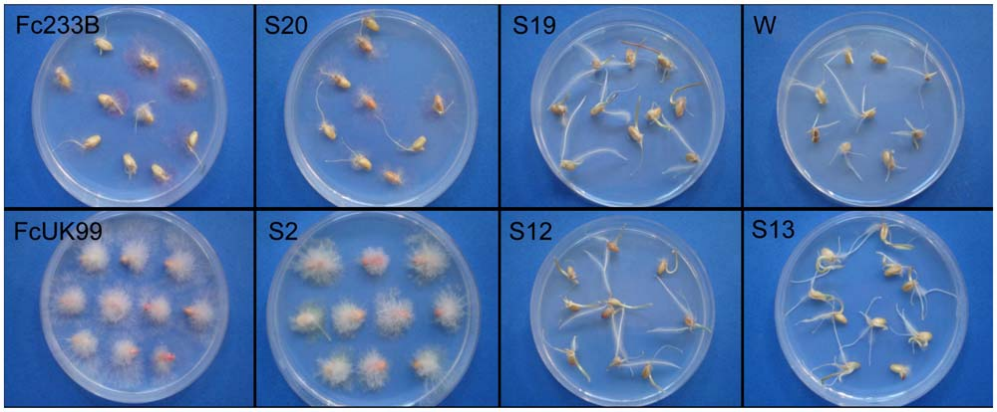
*Fusarium culmorum* wild types and ectopic transformants penetrate into wheat seeds and kill the seedling while Δ*FcStuA* mutants are unable to penetrate the seed. Seed viability after 3 days in the dark at 25°C on water agar (germination) after previous plug inoculation for 3 days with the two wild types (FcUK99 and Fc233B), the mutants (S12, S13 and S19), the ectopic transformants (S2 and S20) and a mock control inoculated with 10 µL water (W). Seeds were washed with NaClO before plating to eliminate external mycelium.

To further investigate the lack of pathogenic behaviour, the pathogenicity test used for FRR was simulated *in vitro* and observed with electron scanning microscopy. As shown in **Fig. 2**, mycelium of the wild-type strain entirely surrounded the seed by forming a complex network of thick and sturdy hyphae, thus preventing germination (**Fig. 2A–2B**), while the developing hyphae of *ΔFcStuA* mutants did not hinder the emergence of the primary root from the caryopsis (**Fig. 2C–2D**), suggesting that the infection leading to FRR in our experimental setting is blocking seed germination before coleoptile growth. This method based on seed emergence *in vitro* gave identical results when compared to *in planta* experiments proving to be as reliable as *in planta* experiments at least for *FcStuA* deletions, suggesting a potential role for general use of *in vitro* testing of FRR-impaired mutants. The *ΔFcStuA* mutants did not kill the seeds and interesting in this assay, the two wild types strains maintained the different level of aggressiveness, namely FcUK99, the highly pathogenic isolate, killing most seeds, and Fc233B, the less pathogenic isolate, killing ∼50% of the seeds (**Fig. 3**). While the wild type hyphae penetrated the seeds, the mutants were unable to kill the seed and were washed away, allowing the seed to germinate freely.

We also observed differences between mutant and wild-type strains in the structure of the mycelium, especially concerning the robustness of hyphae and the capability of the hyphae to colonise a single seed of durum wheat. In addition, compared to both the wild-type and ectopic strains, the *ΔFcStuA* mutants produced fewer aerial mycelium and the hyphae were thin, winding and stunted (**Fig. 2**).

### *FcStuA* deletion induces phenotypic and morphological changes in *F. culmorum*

*FcStuA* deletion mutants grown on PDA showed a phenotype similar to *F. graminearum* mutants [45]. White pigment, sparse mycelium, stunted growth and mycelium embedded in the solid medium were typical phenotypic features of *ΔFcStuA* mutants. Instead, ectopic transformants, that were resistant to hygromycin B, presented a phenotype similar to the respective wild-type strain, namely rapid growth, abundant aerial mycelium, presence of white to yellow or pale orange pigmentation in the media which became brown to red-brown in older cultures.

The mycelial surface of *ΔFcStuA* colonies was characterised by low hydrophobicity unlike wild-type and ectopic strains: the ratio between the time required to absorb a 20 µL drop of water by wild types compared to their respective *ΔFcStuA* strains was approximately 10 for FcUK99 and 3 for Fc233B. In both cases water penetrated into the mycelial surface more quickly in the gene-deletion mutants (**Table 2**).

*FcStuA* deletion mutants grown in liquid CMC produced fewer macroconidia/mL than ectopic transformants and wild-type strains: conidiogenesis of *ΔFcStuA* mutants was reduced by 5 to 10 times (**Table 2**) when compared to original wild-types. *ΔFcStuA* mutants showed a delayed germination of conidia compared to the respective ectopic transformants and wild-type strains at 4 h. However, the effect was minimal at 8 h, with all conidia germinating at a similar level (**Table 2**). The delayed germination of *ΔFcStuA* conidia was not linked to the ability of mycelium to grow. In fact, fungal biomass of the mutants in pectolytic medium was higher when compared to wild types and ectopics (*P*⊕<⊕0.05). The phenomenon was not observed in cultured mycelium after 8 days of growth in toxin- inducing medium, where no significant differences were observed (data not shown).

**Figure 4:**
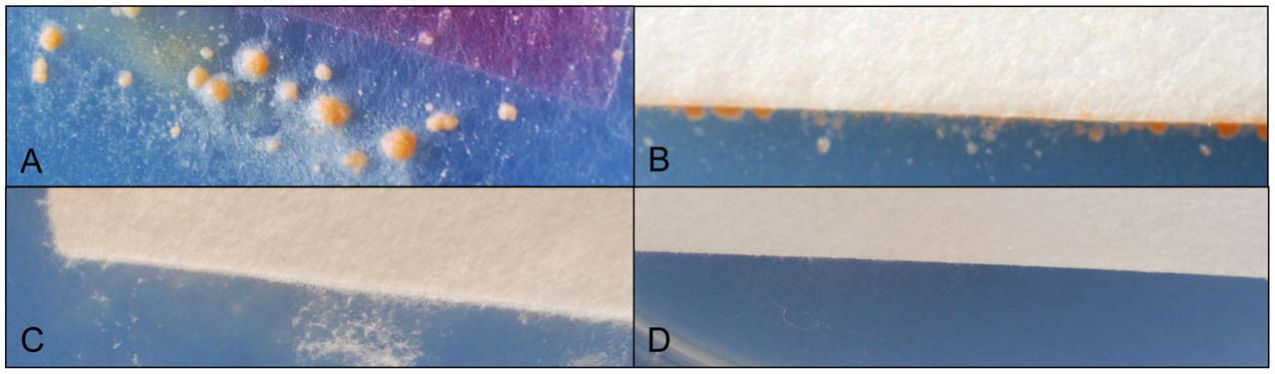
Δ**FcStuA mutants of *Fusarium culmorum* are unable to produce sporodochia. Sporodochia formation on SNA after 18 days growth. A: FcUK99, B: Fc233B; C: S12 (mutant obtained from A); D: S19 (mutant obtained from D).

**Figure 5:**
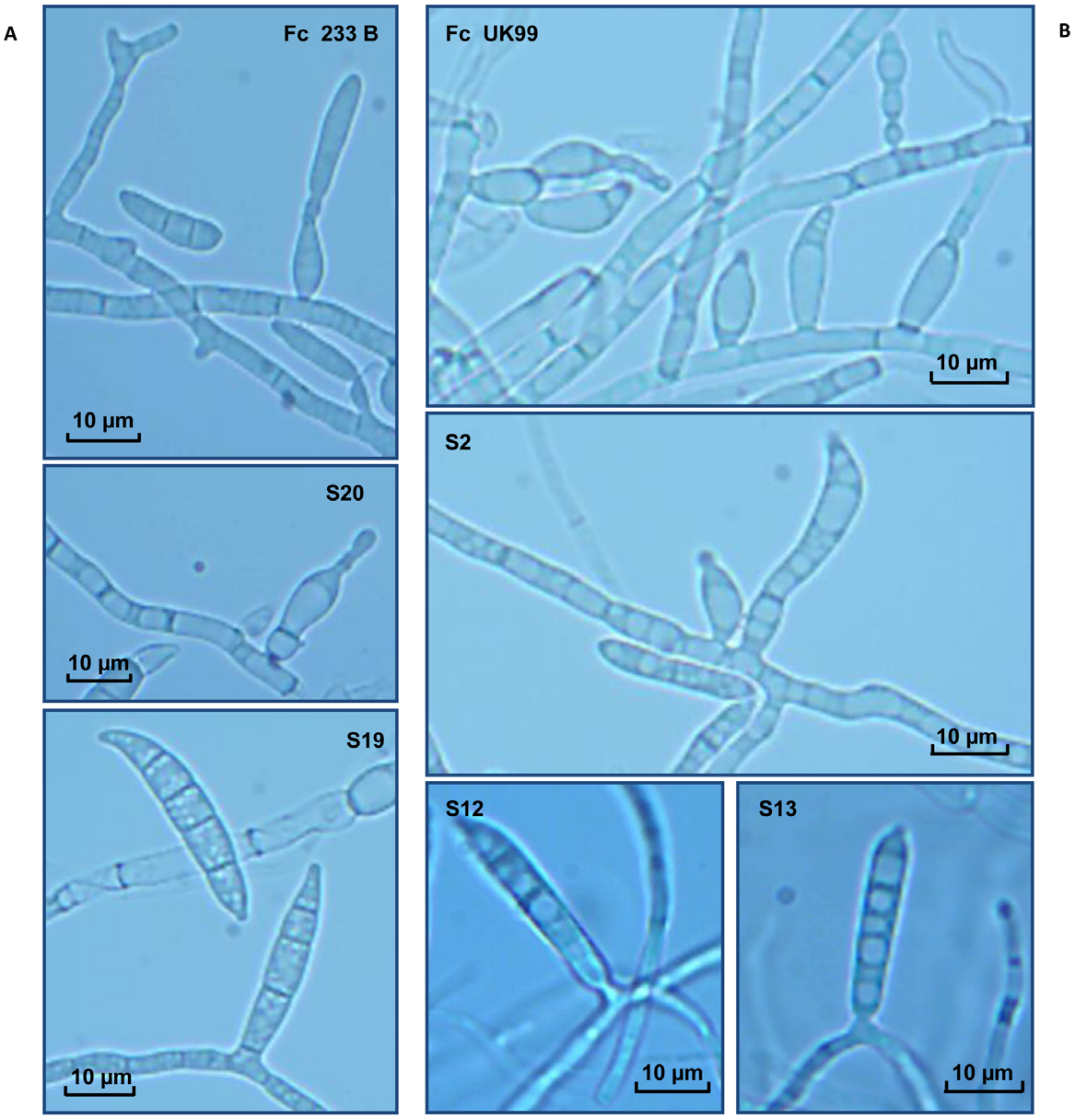
An *in vitro* comparison of the asexual development of the wild-type and Δ*FcStuA* mutant strains of *Fusarium culmorum*. Conidiogenesis by *StuA* deletion mutants, ectopic transformants and wild-type strains was observed with a light microscope (OLYMPUS BX41). Macroconidia of the wild-type FcUK99 strain (A) and the ectopic S20 strain (B) are formed from monophialides on branched conidiophores. Macroconidia of the Δ*FcStuA* mutant strains spores are generated directly from hyphae as in S12 and S13 (*StuA* mutants). (B) The same observations were made for *FcStuA* deletion mutant S19 obtained from strain Fc233B. Photos were taken with MOTICAM 2500 5.O MP live resolution (Motic). Hyphal diameter is smaller in the mutant.

Wild-type strains and ectopic transformants grown on SNA produced abundant sporodochia of orange to brown colour, while *FcStuA* deletion mutants did not form sporodochia (**Fig. 4**). Furthermore, in wild-type strains and ectopic transformants, macroconidia were generated from monophialides on branched conidiophores, while in *FcStuA* mutants, macroconidia were formed directly on slender and twisted hyphae (**Fig. 5**).

### Catalase activity is influenced by FcStuA but not sensitivity to oxidative stress

**Figure 6:**
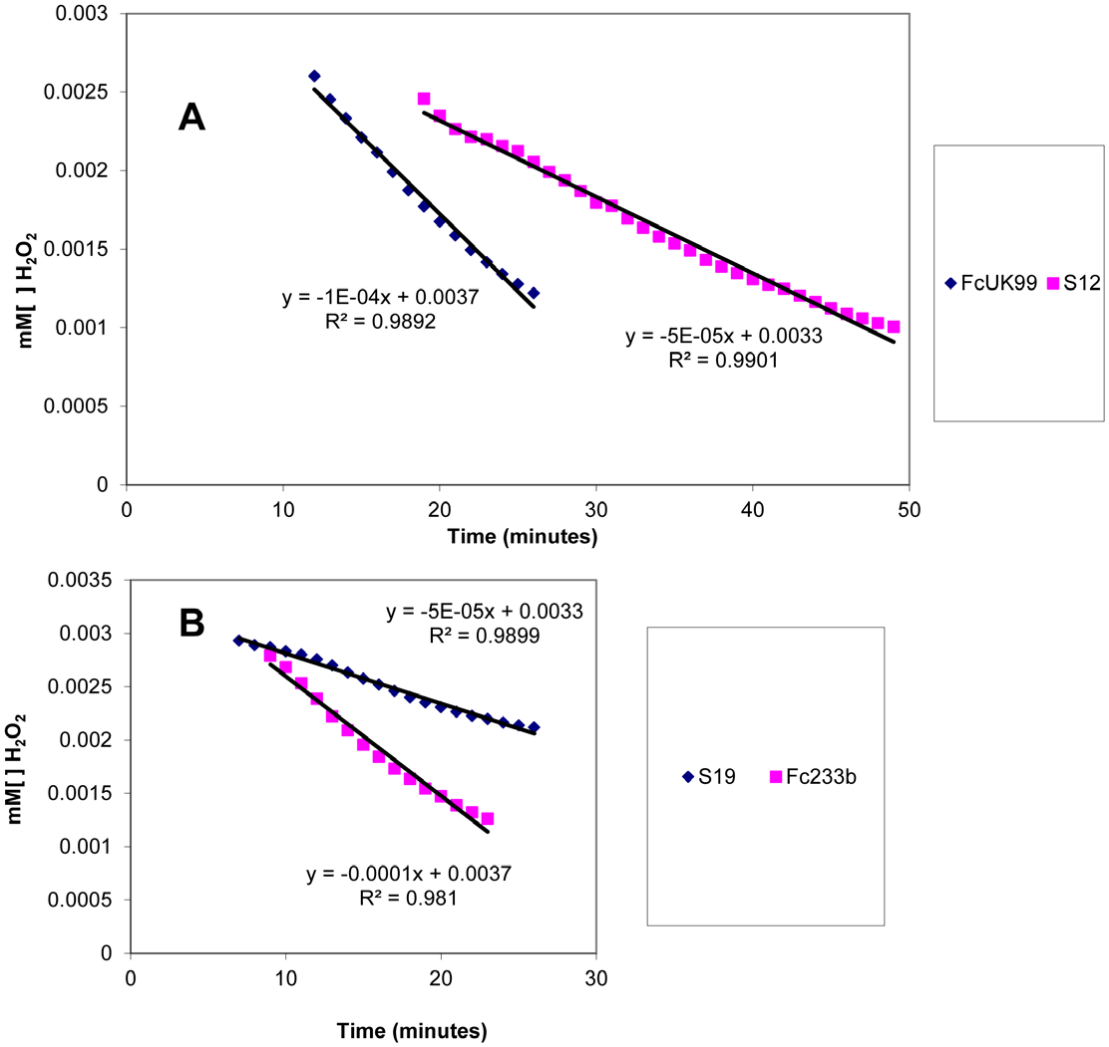
Catalase activity is reduced in the Δ*FcStuA* mutants of *Fusarium culmorum*. Linear phase of degradation of H_2_O_2_ over time inferred by reduced light absorbance at 240 nm. Ten microliter of a spore suspension (10^6^ mL^−1^) of FcUK99 and Δ*FcStuA* S12 (A) and Fc233B and Δ*FcStuA* S19 were suspended in a solution of 3.5 mM H_2_O_2_. Absorbance of the solution was read every minute for a total of 80 min. Results show that wild types catalyse H_2_O_2_ more efficiently than Δ*FcStuA* respective mutants.

The ability of the various strains to cope with oxidative stress was tested by growing fungal colonies on solid agar containing either hydrogen peroxide at 3 mM, potassium persulfate at 50 mM or methyl-viologen at 10 mM and comparing the radial growth ratios at 5 days. Both mutants and wild types were inhibited in a similar manner by the different sources of oxidative stress (data not shown). When mutants and wild type were exposed to increasing concentrations of hydrogen peroxide, both stopped their growth at 5 mM hydrogen peroxide concentration suggesting similar sensitivity to oxidative stress. In the previous *F. graminearum* study [45] a decreased catalase activity of the *stuA* mutants was reported. We therefore investigated the activity of catalase in FcUK99, S12, Fc233B and S19. The decreased activity in both mutants showed by the different slope of hydrogen peroxide degradation curves when compared to the respective wild types confirmed the results observed in *F. graminearum* (**Fig. 6**).

### *FcStuA* deletion results in significant decrease of DON mycotoxin synthesis *in vitro*

The *ΔFcStuA* mutants produced only 5% of the toxin compared of the wild-type FcUK99 and its ectopic strain. Both independent gene deletion mutants tested had a similar decrease of toxin production, suggesting the effective role of FcStuA in controlling toxin synthesis but toxin production was not completely abolished as observed in *F. graminearum* [45], [46].

### Deletion of *FcStuA* gene decreases the production of pectolytic enzymes significantly

In order to further investigate the mechanism leading to the lack of pathogenicity that are not depending on toxin production we applied an inhibition assay on agarose Petri dishes (cup plate). This assay was used to estimate and compare the activity of pectin matrix degradation in *FcStuA* mutants (S12–S13 and S19), ectopic transformants (S2 and S20) and their wild-type strains (FcUk99 and Fc233B). The polygalacturonase enzyme activity was found to be significantly different between the various non-pathogenic *ΔFcStuA* mutants and their respective virulent wild-type control strains (**Table 2**). This suggests that the *FcStuA* gene is involved in the mechanisms of polygalacturonic enzyme secretion during infection and colonisation of the host.

### Δ*FcStuA* mutants have an altered glycolytic metabolism and are able to degrade various carbon sources to different extent

**Figure 7:**
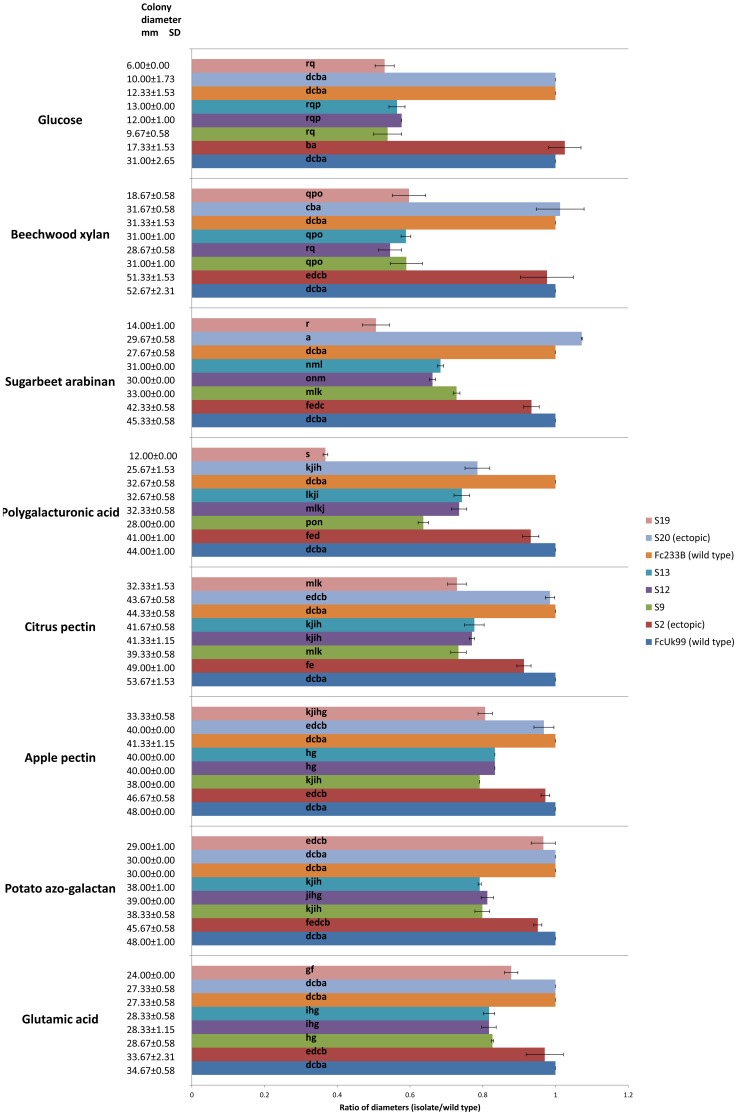
Growth of wild types, mutants and ectopic strains on different carbon sources obtained from various plant origins. Colony diameter in mm and standard deviation are indicated on the left and refer to the average of 3 biological replicates. The graph indicates the effect of carbon source on the growth ratio (mutant / wild type) after 5 days of growth on Petri dishes containing glucose, beechwood xylan, sugar beet arabinan, polygalacturonic acid, citrus pectin, apple pectin, potato azo-galactan or glutamic acid as a sole carbon source. Bars labelled with the same letter are not significantly different according to Tukey's post hoc test p<0.05. Bars represent SDs of the ratios (within each strain) of three biological replicates.

To further verify that the impairment in polygalacturonase enzyme production was due to the activity of StuA controlled genes, different substrates from various plant origins were used as the sole carbon source. Growth diameter after 5 days ranged from 6 mm to 56 mm depending on the strain and the carbon source (**Fig 7**). Highly significant (p<0.0001) effects of type of carbon source and fungal strain as well as a highly significant interaction of carbon source x fungal strain on fungal colony diameter were observed. The growth of all mutants was reduced to about 80% of the wild type strain when glutamic acid was the sole carbon source, whereas the growth of the corresponding ectopic mutants was not significantly different from the wild type (**Fig. 7**). The use of potato azo-galactan, apple pectin and citrus pectin resulted in a comparable growth similar to that observed with glutamic acid. The growth of all mutants was reduced to about 55% of the wild type strain when glucose was the sole carbon source, whereas the growth of the ectopics was again not significantly different from the wild type (**Fig. 7**). Beechwood xylan had a similar effect to glucose. The response of the mutants towards sugarbeet arabinan and polygalacturonic acid was variable and somewhere between the low relative inhibition level observed for glutamic acid and the high relative inhibition level observed for glucose (**Fig. 7**).

### Fungicide sensitivity of *ΔFcStuA* mutants is not altered significantly

**Figure 8:**
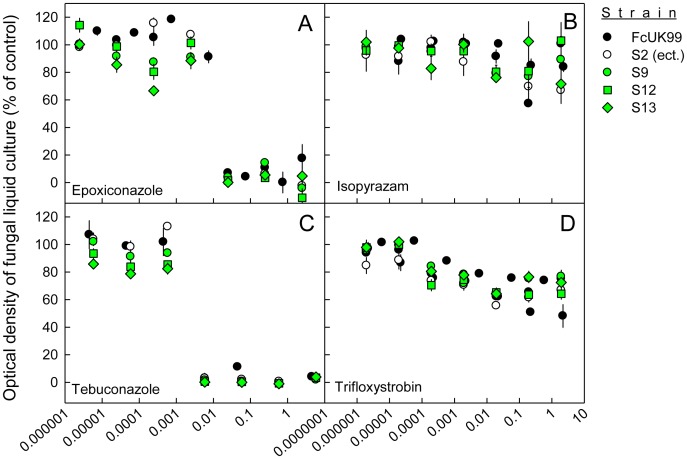
The sensitivity of the highly pathogenic wild-type FcUK99, ectopic and *FcStuA* deletion mutants to various fungicides. The optical density of liquid cultures of the *Fusarium culmorum* wild type strain FcUK99, an ectopic and *FcStuA* deficient strains (S9, S12, S13) growing in 200 µL of 12.5% (w/w) potato dextrose broth when augmented with different concentrations of fungicides (A) epoxiconazole, (B) isopyrazam, (C) tebuconazole, or (D) trifloxystrobin. The optical density measurements were made after 5 days of incubation at 120 rpm and 22°C in the dark. An optical density close to 100% indicates no sensitivity to the fungicide, whilst an optical density close to 0% full inhibition. Error bars represent the standard error of 3 replicates.

**Figure 9:**
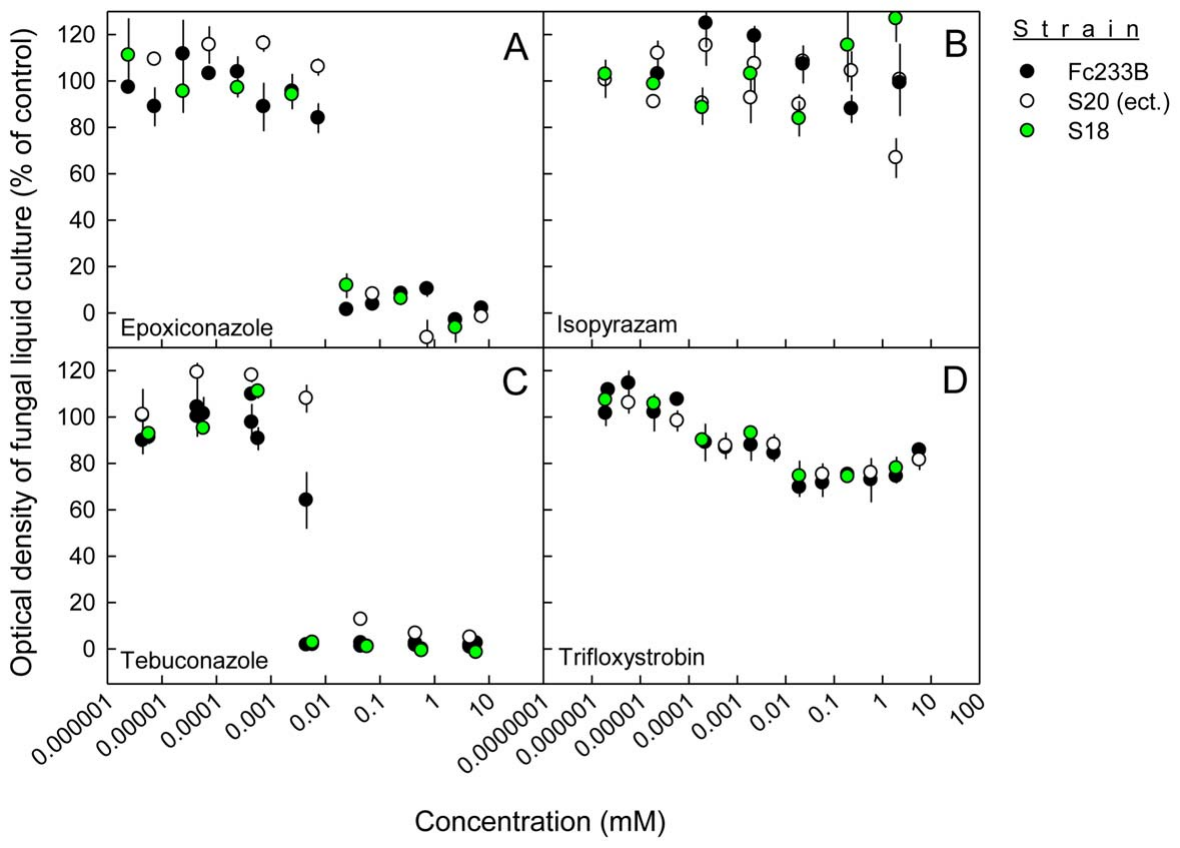
The sensitivity of the mildly pathogenic wild-type Fc233B, ectopic and *FcStuA* deficient strains to various fungicides. Optical density of the *Fusarium culmorum* wild-type strain Fc233B, an ectopic (S20) and a *FcStuA* deficient strain (S19) in liquid cultures (200 µL, medium: 12.5% (w/w) potato dextrose broth) as affected by epoxiconazole (A), isopyrazam (B), tebuconazole (C), or trifloxystrobin (D) concentration after 5 days of incubation at 120 rpm and 22°C in the dark. An optical density close to 100% indicates no sensitivity to the fungicide, whilst an optical density close to 0% full inhibition. Error bars represent the standard error of 3 replicates.

The demethylase inhibitors epoxiconazole and tebuconzole inhibited *F. culmorum* FcUK99 by 50% at concentrations of 0.0142 ± 0.0030 and 0.0041 ± 0.0012 mM, respectively (**Fig. 8A, C**). By contrast, the complex II and complex III respiration inhibitors isopyrazam and trifloxystrobin were unable to inhibit *F. culmorum* FcUK99 and Fc233B up to a concentration of 1 mM (**Fig. 8B, D**). The ectopic strains S2 and S20 expressed the same fungicide sensitivity profile as the wild type strains (**Fig. 9**). The fungicide sensitivity of *FcStuA* deficient mutants was similar to the sensitivity of the wild type strain and the ectopic transformants (**Fig. 8** **,** **9**).

## Discussion

This work reports on the identification of a pathogenicity factor in *F. culmorum* that adds to the very few other genes known to play a role in the pathogenic behaviour of this species [47], [48]. Besides the genes shown to contribute to trichothecene production [19], this is only the second functionally characterised pathogenicity gene involved in foot and root rot disease caused by a *Fusarium* species [42].

### *FcStuA* gene affects a large set of phenotypic characters

APSES proteins, homologues of StuAp from *Aspergillus nidulans* [49], were reported to control metabolic, morphological and developmental stages in various fungal species (for details see: [50]). This was confirmed in *F. culmorum* where FcStuA controls both conidial germination confirming that budding factors are influenced by this gene [51] and spore production, influences the growth of aerial mycelium, the hydrophobicity of the mycelium and its thickness, having overall a significant negative effect on the fitness of the strains lacking the gene. The StuA protein is necessary for the production of conidiophores and phialides as shown previously [45], [51], [52]. The direct conidiation from hyphae is probably the main reason for the reduced number of conidia produced by the mutants.

The *FcStuA* gene sequence has a very high homology to the *F. graminearum FgStuA* gene. The lack of significant amino acid differences between the two sequences indicated an identical structure of the two proteins. When comparing the similarity of *FgStuA* and *FcStuA* to a general level of similarity between the two species, at least with partial data obtained from a transposon tagging approach [53], the level of conservation seems to be higher than average: 98.5% vs 96%. It is therefore suggested that *FcStuA* is under strong selective pressure having a crucial role in cell regulation and therefore being highly conserved between the two closely related species.

The morphological effects of the deletion of *StuA* in the two species were found to be highly overlapping. However, in this study and another [48], specific characteristics were identified that differed between these two species. Identifying differences and then their underlying genome basis will help to elucidate the genes which play a role in the adaptation and fitness of the two phytopathogens. For example, the overall reduction of conidia production in the *StuA* mutants was quantitatively different between *F. graminearum* (1:10000 drop) and *F. culmorum* (1:10 drop). This difference suggests that in *F. culmorum* compensation mechanisms and / or an alternative signalling pathway(s) for spore production exist.

Few regulators are known to control secondary metabolites production in fungi [54]. FcStuA was confirmed to regulate secondary metabolite production in *F. culmorum*. While pigment production was absent in both species suggesting that aurofusarin and other pigments were not produced in the *StuA* mutants, the level of deoxynivalenol (DON) mycotoxin production *in vitro* was reduced differently between *F. culmorum* and *F. graminerum*. In *F. graminearum* toxin production was either zero or below 1% of wild-type levels [45], [46] while in the present study *F. culmorum* mutants produced 5% of DON, when compared to wild-type. The *F. graminearum* strain PH-1 is a DON and 15ADON producer, whereas FcUK99 is a DON and 3ADON producer [21]. It is possible that these chemotype differences and/ or the media used to induce DON production *in vitro* conditions may partially explain the species differences observed between the two studies. Nonetheless, overall *FcStuA* gene deletion seems to have milder effects compared to the *FgStuA* gene deletion on DON mycotoxin production *in vitro*.

Despite numerous attempts, it was impossible to complement the wild-type gene back into the *ΔFcStuA* mutants. Direct co-transformation with the gene and a plasmid carrying geneticin resistance [34] was unsuccessful as well as the attempt to clone the gene in a bacterial strain. The same phenomenon was observed on *FgStuA* gene of *F. graminearum* [45], as well as for *U. maydis* [55] and *Stagonospora nodorum* [56]. Despite the lack of available *StuA* complemented *F. culmorum* strains for comparative purposes, we are confident that all the effects presented here are solely due to the lack of the FcStuA transcription factor for the following two reasons: (1) all mutants showed the same phenotype, while both ectopic strains were similar to respective wild-types, (2) the effects of *FcStuA* gene loss were confirmed in the genetic background of two *F. culmorum* strains with different geographic origins and different mycotoxin chemotypes.

### How could FcStuA control pathogenicity on wheat?

The identification and functional characterisation of a transcription factor containing a highly conserved bHLH-like APSES domain [6] in *F. culmorum* was carried out in order to investigate the role of the gene in a previously untested pathogenicity mechanism during seed germination/initial seedling plant establishment. The gene is known to be a pathogenicity factor for many fungal species, including *F. graminearum* [45] and wheat heads, *Ustilago maydis* and maize leaves [55], *Glomerella cingulata* and apple fruit [57] and *Stagonospora nodorum* and wheat leaves [56]. These fungi are not taxonomically closely related and also have diverse *in planta* lifestyle including biotrophy, hemibiotrophy and necrotrophy.

In comparison, in the taxonomically more closely related species *F. oxysporum* f. sp. *lycopersici*, causal agent of root rot of tomato, the *FoStuA* deletion mutant was not affected in its virulence towards tomato plants and root and stem tissues [52]. Given the different role(s) played by StuA homologues in *F. graminearum* and *F. oxysporum* (the two most closely related organisms to *F. culmorum*) we investigated the effect of the gene in *F. culmorum* combining two different cereal pathosystems (head infection and foot and root rot infection of wheat) and three non-cereal hosts (tomato fruit, apple fruit and potato tuber). The lack of symptoms following inoculation with each of the *ΔFcStuA* mutants tested proved that FcStuA is essential for colonisation of the tissues in all five plant cases. By comparing the pathogenic behaviour of *FcStuA* mutants obtained from a toxin producer and a non-toxin producer strain it was possible to infer a role for DON toxin in pathogenicity assays. In both genetic backgrounds, a loss of pathogenicity was obtained when *FcStuA* was deleted and mild symptoms were caused by the wild type non-toxin producer strain on wheat heads and on wheat seedlings confirming that DON production may not be essential for causing FRR symptoms [20]. Interestingly, the potential ability to produce small amounts of toxin in the FcUK99 *ΔFcStuA* mutant did not induce significant symptoms, further suggesting that the lack of pathogenicity towards both germinating seeds and wheat floral tissue is stopped by other mechanisms directly and / or indirectly controlled by the FcStuA protein. This conclusion is further supported the environmental Scanning Electron Microscope (envSEM) observations. The images obtained revealed the inability of the *ΔFcStuA* mutant to consistently attach on the seed and inhibit seed germination while it is known that toxin is important during the later stage of infection.

Virulence is a highly complex character [58] that can be influenced by different molecular mechanisms. Therefore we attempted to decipher which mechanism could be controlled by FcStuA. Several morphological differences caused by deletion of *FcStuA* that may impact on disease severity were observed. These included slow growth on solid medium as well as reduced colonisation of the cut surfaces of apple, potato and tomato tissues and on the wheat seed surface. On the contrary growth rate was not decreased in liquid medium. Reduced expression of hydrophobins [59] as observed in *F. graminearum* and *Glomerella cingulata* [45], [57] may indeed explain the difference of growth in different media and have a direct effect on the ability to cause disease [60]. To further investigate the possible cause(s) for the inability of *ΔFcStuA* mutants to colonise wheat seeds and colonise wheat stem base tissue efficiently in both parental backgrounds, polygalacturonase enzyme production was investigated. In several other pathosystems, this enzyme activity is considered to play a crucial role in penetration of plant cells and therefore to influence early pathogenic behaviour [61], [62], [63], [64], [65]. By calculating polygalacturonase production per unit dry mass of mycelium, we could correlate total polygalacturonase activity to the amount produced per gram of mycelium. Both types of Δ*FcStuA* mutants produced a lower amount of enzyme. However, a reduced polygalacturonases activity in the mutants is unlikely to be the main factor determining decreased pathogenicity. For example, efficient growth of the Δ*FcStuA* mutants was observed *in vitro* on the different pectin-like carbon sources from apple and citrus as well as on azo-galactan from potato whereas the mutants were unable to efficiently colonise apples and potato slices. While we could confirm that glycolytic process is inhibited in the *StuA* mutants, pectolitic enzymes necessary to degrade the complex set of pectin –like compounds [66], as tested in this study, are only partially regulated (directly or indirectly) by *FcStuA* gene such as xylan degrading enzymes. Therefore it is confirmed that pectin enzyme sets are controlled by a diverse set of regulatory circuits [67]. Catalase activity was also significantly decreased in the Δ*FcStuA* mutant tested here confirming previous results produced for the corresponding *FgStuA* mutants [45]. Δ*FcStuA* mutants response to the oxidative stress does not differ from the wild type excluding that, during plant infection and colonisation, the fungal cells lacking StuA cope less efficiently with oxidative stress resulting from plant defense mechanisms. Therefore it is probable that the decreased catalase activity does not have an influence on the decreased pathogenicity of the mutants. Due to the reduced DON production by the Δ*FcStuA* mutants, minimisation of the induction of plant defense responses, via inhibition of host protein translation, is likely to be low. It can be that the reduced catalase activity and the inability to produce toxin belong to the same molecular pathway linking environmental sensing and toxin production [68], [69], [70] in the fungus and that FcStuA is controlling genes upstream in this pathway.

It is not possible to pinpoint which compromised mechanism or combination of compromised mechanisms is responsible for the initial lack of seed penetration and / or young seedling colonisation. However, from the phenotypic data presented here, the lack of the StuA protein in *F. culmorum* causes the very early arrest of the infection process.

### FcStuA as a potential target for novel chemicals

The functional characterisation of genes controlling pathogenicity and developmental stages on fungal plant pathogens is an important strategy for potential development of novel molecules able to control pathogen growth and infection. Indeed, cellular controllers of fungal fitness, possibly specific to the fungal domain as APSES proteins [50], are ideal targets for the design of new fungicides [71].

As shown here, FcStuA is controlling pathogenicity *via* a set of different mechanisms that are not exclusively linked to the toxin production making it a perfect target for novel molecules with a fungistatic activity combined with a toxin blocking production activity. Given the emergence of resistance phenomena, the use of novel fungicide combinations and the adoption of multiple active compounds, is the most efficient solution at the moment [72]. It was therefore of interest to know if putative molecules that target FcStuA transcription factors, simulated here *via* the complete elimination of the gene, may have any negative effect on the onset of inhibition to specific classes of fungicides [73]. Therefore we studied the level of resistance by the mutants to three classes of fungicides widely used in agriculture. *Fusarium* species are partially sensitive towards fungicides belonging to the group of demethylase inhibitors such as epoxiconazole and tebuconazole, but are intrinsically resistant towards complex III respiration inhibitors such as trifloxystrobin [44]. We observed no significant effect (in both direction: i.e. resistance or susceptibility) caused by the deletion of the FcStuA transcription factor suggesting that the protein is not directly involved in controlling the processes of intrinsic fungicide resistance to strobilurins or partial susceptibility to azoles. It is noteworthy that the two wild type *F. culmorum* strains tested for growth in the presence of the respiration inhibitor isopyrazam, a recently introduced compound revealed that this species is also intrinsically resistant to complex II inhibitors as already observed in *F. graminearum* [74].

Our result suggests that a putative molecule able to target the specificities of the FcStuA protein, would not increase the risk of resistance against other known fungicides. Given the restricted spectrum of available molecules against *Fusarium* species it seems promising to suggest APSES proteins as novel targets for developing new pathogen controlling measures.

## Conclusions

Our work presented evidence, for the first time in *F. culmorum*, that FcStuA controls several pathways within the fungal cell that directly or indirectly regulate morphological development, the glycolytic process, catalase activity, exopectinase and pectin like metabolism and trichothecene mycotoxin production. By using two strains differing for virulence and toxin production we were able to show that pathogenic behaviour is not simply determined by the lack of toxin production in Δ*FcStuA* mutants. As in other plant pathogenic fungi able to produce toxins that are involved in the pathogenic process (*F. graminearum*, *S. nodorum, U. maydis*) pathogenicity was impaired, while in *F. oxysporum* - a species not yet known to produce toxin-like pathogenicity factors - the pathogenic process was not altered. We therefore investigated the role of toxin production in determining *F. culmorum ΔFcStuA* pathogenicity. Our data suggest that the toxinogenic process is not the determinant of pathogenicity impairment in the deletion mutant. We showed that mechanisms that may determine the lack of pathogenicity in *FcStuA* mutants result in early blocking of the infection process. Decreased conidial germination efficiency (hence decreasing the inoculum) and reduced robustness of mycelium probably determine the inability to overcome plant defense mechanisms by the fungus.

Future studies focusing on the interaction of this transcription factor with other regulatory proteins may shed light on the regulatory pathways that are involved in shaping the different aspects of the fungual phenotype. As a molecule targeting the StuA protein would induce very low selective pressure (no biocide activity) but would decrease virulence and toxin production without increasing the risk of resistance to other known fungicides, the exploration of compounds able to interact with this protein are of interest for developing novel control strategies.

## Supporting Information

Figure S1 **(A)** *FcStuA* gene deletion procedure. The name and location of each primer used in split marker recombination are marked (scheme adapted from [30]). (**B**) Agarose gel electrophoresis of PCR products obtained from *StuA* mutant strain (S19), ectopic transformant (S20) and wild-type strain (Fc233B). Primers stuA NF and stuA NR were used to verify the *FcStuA* gene deletion. The *ΔFcStuA* mutants lacked the PCR band of 401 bp corresponding to the endogenous *FcStuA* gene, while this fragment was amplified in the wild-type strain (pink box) and in ectopic transformant (green box). The primers ITS1 and ITS4 were used as an internal control for DNA quality. The gel on the right shows results of PCR analysis with the following primer pairs: *StuA* 1F-*StuA* 4R, *StuA* 1F-*StuA* NR, *StuA* NF-*StuA* 4R used to confirm the deletion of *StuA* gene. (**C**) Independent confirmation of the specific deletion of the *FcStuA* gene sequence was achieved by Southern blot analysis of 11 *StuA* deleted transformants. Genomic DNAs were digested with *Eco*RV and after transfer of the DNA to the membrane, the hybridisation was done by labelling a partial gene *stuA* specific probe (401 bp). The fragment sizes expected were: 3,400 bp and 2,240 bp, respectively, for *ΔFcStuA* mutants and for control (wild-type strains and ectopic transformants).
(TIF)

Figure S2 **Alignment of FcStuA protein with homologues obtained from three other** ***Fusarium*** **species.** The sequence is 98.5% identical to *F. graminearum* protein (FGSG_10129) with the 2 amino acid differences not linked to any particular functional role. The blue arrow shows the conserved APSES domain.
(TIF)
