# Supplementary figures and images for: FcStuA from Fusarium culmorum Controls Wheat Foot and Root Rot in a Toxin Dispensable Manner
Source: PLoS One. 2013 Feb 22;8(2):e57429. doi: 10.1371/journal.pone.0057429 (PMC3579838; doi:10.1371/journal.pone.0057429)

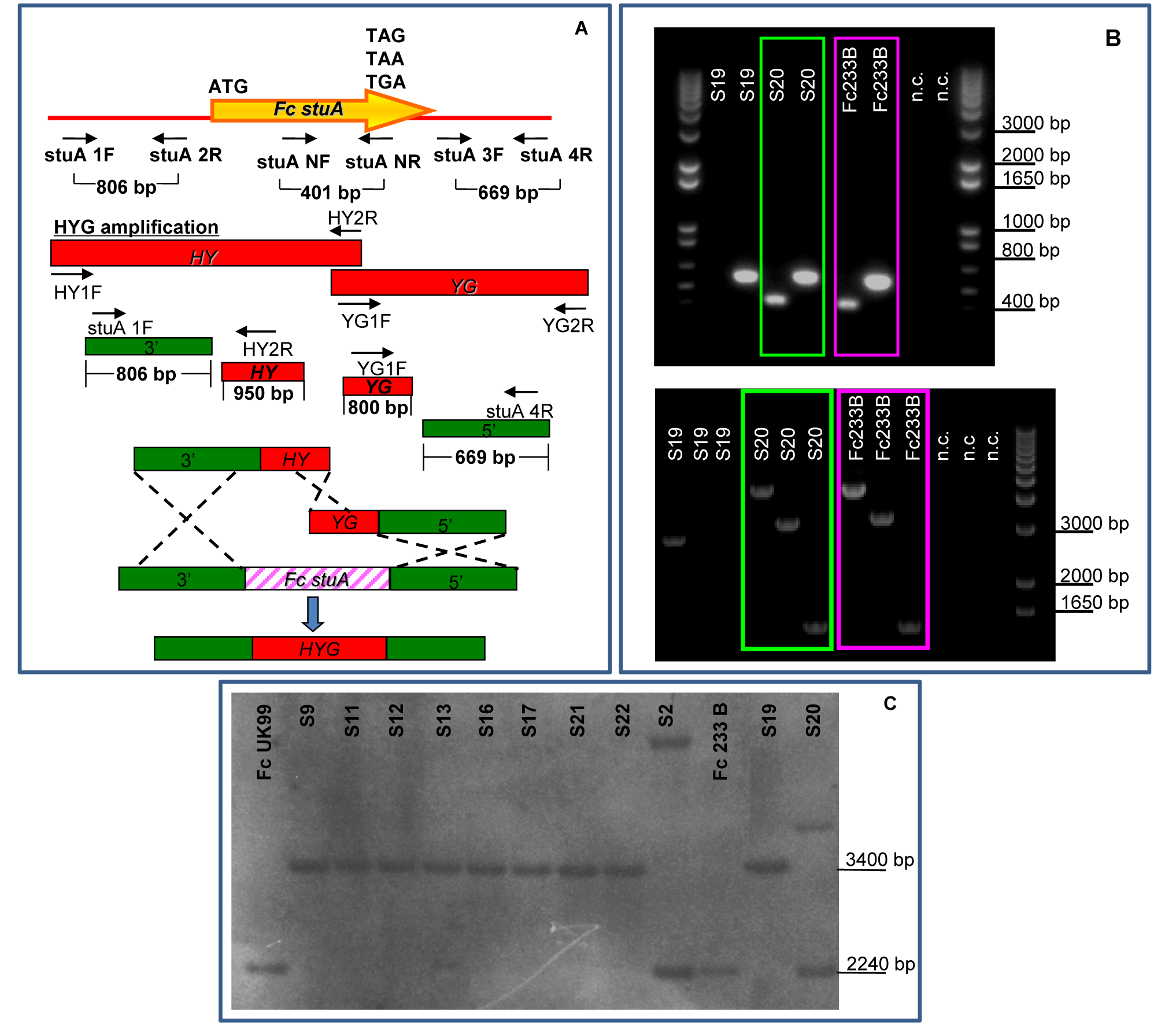

Supplement: Figure S1 — (A) FcStuA gene deletion procedure. The name and location of each primer used in split marker recombination are marked (scheme adapted from [30]). (B) Agarose gel electrophoresis of PCR products obtained from StuA mutant strain (S19), ectopic transformant (S20) and wild-type strain (Fc233B). Primers stuA NF and stuA NR were used to verify the FcStuA gene deletion. The ΔFcStuA mutants lacked the PCR band of 401 bp corresponding to the endogenous FcStuA gene, while this fragment was amplified in the wild-type strain (pink box) and in ectopic transformant (green box). The primers ITS1 and ITS4 were used as an internal control for DNA quality. The gel on the right shows results of PCR analysis with the following primer pairs: StuA 1F-StuA 4R, StuA 1F-StuA NR, StuA NF-StuA 4R used to confirm the deletion of StuA gene. (C) Independent confirmation of the specific deletion of the FcStuA gene sequence was achieved by Southern blot analysis of 11 StuA deleted transformants. Genomic DNAs were digested with EcoRV and after transfer of the DNA to the membrane, the hybridisation was done by labelling a partial gene stuA specific probe (401 bp). The fragment sizes expected were: 3,400 bp and 2,240 bp, respectively, for ΔFcStuA mutants and for control (wild-type strains and ectopic transformants). (TIF) [file pone.0057429.s001.tif]

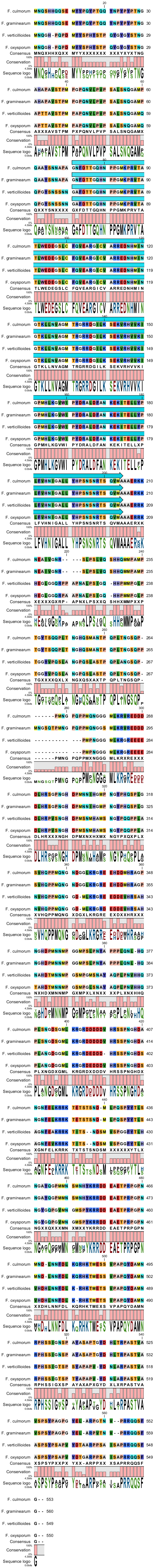

Supplement: Figure S2 — Alignment of FcStuA protein with homologues obtained from three other Fusarium species. The sequence is 98.5% identical to F. graminearum protein (FGSG_10129) with the 2 amino acid differences not linked to any particular functional role. The blue arrow shows the conserved APSES domain. (TIF) [file pone.0057429.s002.tif]
